# Clinical application of cfDNA: moving in the right direction, but still a long way to go

**DOI:** 10.18632/oncotarget.26224

**Published:** 2018-10-16

**Authors:** Shehara Mendis, Jonathan M. Loree

**Affiliations:** Jonathan M. Loree: Department of Medical Oncology, BC Cancer, Vancouver, British Columbia, Canada

**Keywords:** colorectal cancer, ctDNA, BRAF, next generation sequencing, non-melanoma

In Ahlborn et al’s recent assessment of the utility of cell-free DNA (cfDNA) to track tumor responses, they focus on a subset of 23 BRAFV600E-mutated non-melanoma tumors identified within the 455 patients enrolled on the Copenhagen Prospective Personalized Oncology (CoPPO) Program [1]. These were predominantly colorectal cancer (CRC) patients (16/23), but also included 7 non-colorectal patients (bile duct, lung and pancreatic). Patients underwent pre-treatment biopsy and cfDNA was collected every 4 weeks. Seventeen of the 23 patients were treated with BRAF/MEK inhibition, BRAF/EGFR inhibition or BRAF/EGFR inhibition with chemotherapy. The median progression free survival (PFS) of 4.8 months and overall survival (OS) of 15 months shown here in a CRC-predominant cohort is comparable to other studies in CRC using BRAF inhibition in combination with similar agents [2, 3].

Ahlborn et al show that baseline cfDNA and baseline circulating mutant BRAFV600E DNA (ctBRAF^V600E^) was modestly correlated with tumor burden, and that baseline ctBRAF^V600E^ mutant fraction ≥0.05 trended towards shorter PFS and OS. More importantly, the authors demonstrate that serial cfDNA measurements can predict early progression and a lack of benefit from targeted therapy. They found that a reduction in ctBRAF^V600E^ of <50% at 4 and 12 weeks correlated with worse survival and that an increase in ctBRAF^V600E^ by ≥50% preceded radiological progression in 11/14 cases that progressed. These findings match those of Corcoran et al, who showed that depth of change in ctBRAF^V600E^ correlated with depth of radiologic response and that rising ctBRAF^V600E^ could be used to predict progression [2]. This model of using changes in cfDNA over time has been used to precisely predict time to progression in patients with non-BRAF mutations, and highlights firstly the utility of cfDNA to track clonal dynamics in patients with diverse driver mutations and biology and secondly its potential role as a predictive biomarker [4].

BRAF mutations occur in 5-10% of non-melanoma cancer [5] and are frequently a driver mutation. BRAF-mutated colorectal cancer (CRC) in particular is associated with a poor prognosis. Despite encouraging results in early trials [3, 6], BRAF inhibition in CRC has failed to achieve the same levels of efficacy seen in melanoma. Acquired resistance to BRAF directed therapy is often driven by genomic alterations that reactivate MAPK signalling [7]. Despite the ease of drawing plasma to assess cfDNA, there are still significant costs to these assays and the optimal interval between serial assessments for providing clinically actionable information remains undear. At present, until further strategies to block MAPK resistance mechanisms are developed, frequent samplings benefit us by demonstrating the diversity of genomic alterations that may drive resistance, but do little to help the patients in front of us.

Furthermore, the depth and breadth of sequencing platforms is relevant to navigation of the cfDNA landscape. The digital droplet PCR (ddPCR) method used here for the majority of the study is a focussed method of sequencing, which tests for abnormalities in a single gene at specific locations. It provides ultra-sensitive quantification but lacks the breadth of sequencing needed to identify arising resistance mechanisms that occur in many genes. The investigators paired ddPCR with cfDNA exome sequencing for this end. This highlights a major challenge in adopting cfDNA technologies: what is the optimal design that balances assay depth for tracking disease response with assay breadth to identify emerging resistance mechanisms [1, 4]?

Even when depth and breadth of sequencing are adequate, the challenge remains of interpreting clinical and genomic information in uncommon variants or exceptional responders [8, 9]. Ahlborn et al found 6 novel MAPK-related variants in plasma samples collected at progression. Are these variants true oncogenic drivers or simply passenger mutations? Caution is needed in even the assumption that all mutations in cfDNA are of tumor origin, as evidenced by Strickler et al finding JAK2^V617F^ mutations in 1% of their CRC cfDNA cohort at baseline, which more likely originated from haemopoietic clones of unclear significance rather than CRC [10].

Despite being a small, heterogenous study, Ahlborn et al have demonstrated that by tracking ctBRAF^V600E^ change from baseline at 4 and 12 weeks, we have a clinical biomarker for patients most likely to have durable benefit from BRAF inhibition. In addition, rising ctBRAF^V600E^ was associated with worse outcomes and may be an early warning of treatment failure and a need to change strategies. This is of immense utility in patients who historically do very poorly. Furthermore, 2 of 3 patients with undetectable levels of mutant BRAF throughout this study experienced prolonged survival, adding to the body of evidence suggesting that baseline non-detectable cfDNA in CRC correlates with improved survival [4].

These findings leave us with many unanswered questions for future studies. For those that initially respond to therapy, given that subsequent increases in ctBRAF^V600E^ foreshadow radiologic progression, what are the implications of these findings for clinical management? Should we tailor frequency of imaging to changes in ctBRAF^V600E^? Should we change treatment at the time of ctBRAF^V600E^ “escape”, or based on mathematical modeling predictions rather than waiting for radiological progression [4]? These are important questions given the experience in BRAF mutant CRC is that of rapid progression and decline in performance status that often precludes further lines of treatment. And beyond that, upon withholding BRAF directed therapy, if clinically detectable variants conferring resistance then fade away, could we reintroduce BRAF targeted therapy at that point and expect to derive benefit?

In the meantime, until we become more sophisticated at inhibiting BRAF-mediated oncogenesis, accurate upfront identification is needed of patients unlikely to respond to our current armamentarium against BRAF-mutant tumors, to spare such patients potentially futile treatment. Khan et al have demonstrated that in RAS wild-type patients, detecting baseline aberrations in resistance pathways can predict those refractory to EGFR inhibitors [4]. In a similar vein, much more work needs to be done to understand mechanisms of resistance to targeted therapy in BRAF-mutated CRC and how to incorporate cfDNA into clinical decision making. Despite recent rapid advances in the field of personalized oncogenomics, we have a long way to go before bringing this to the clinic.

## References

[1] Ahlborn LB (2018). Oncotarget.

[2] Corcoran RB (2018). Cancer Discov.

[3] Kopetz S (2017). J Clin Oncol.

[4] Khan KH (2018). Cancer Discov.

[5] Zehir A (2017). Nat Med.

[6] Kopetz S (2015). J Clin Oncol.

[7] Ahronian LG (2015). Cancer Discov.

[8] Lou E (2017). J Natl Compr Canc Netw.

[9] Loree JM (2017). J Natl Compr Canc Netw.

[10] Strickler JH (2018). Cancer Discov.

